# Chicago Society of Ophthalmology and Otology

**Published:** 1886-01

**Authors:** 


THE CHICAGO SOCIETY OF OPHTHALMOLOGY AND OTOLOGY.

The Society convened on October 13th, 1885. F. C. HOTZ, M. D., in the chair. The Society listened to the following paper by DR. LYMAN WARE:

ON OSSIFICATION OF THE CHOROID.

Although calcareous formations within the eye are of frequent occurrence, osseous formations are not so common, but the following case may be instructive, both pathologically and clinically. For a long time all hardened deposits within the eye were considered calcareous and the formation of true bone was much doubted. Knapp collected and studied quite a number of cases of ossifications within the eye, which he published in the Archives of Ophthalmology and Otology, vol. II., p. 1–35.

At that time he considered the capillary layer of the choroid the origin of all intraocular ossification. Since then a number of cases have been reported,* in which they occurred in other structures. The formation of bone is usually preceded by

*Knapp's Archives of Ophthalmology, Volumes VI. and IX.

prolonged and frequently severe intraocular inflammation, and is generally of traumatic origin. The development of bone requires no antecedent conditions other than a vascularized connective tissue. The following is a brief history of a case that came under my observation:

Mr. G. D., farmer, aged twenty, American. When nine years of age he was struck accidentally in the right eye with the handle of a pocket-knife, which completely and instantly destroyed vision and caused severe inflammation in and about the eye, accompanied by great pain and much distension of the ball and surrounding tissues, continuing for several weeks. During the five or six subsequent years the injured eye caused little or no trouble. During the winter of 1875, when he was sixteen years of age, or seven years after the accident, as a result of severe exposure, the injured eye again became painful, at times better, at times worse. Pain usually subsided in a few days if the patient remained quiet in a darkened room and made frequent hot applications to the eye. In the winter of 1880, the left eye began to be sensitive to the light, particularly to artificial light or reflected light from snow.

The patient was first seen in July, 1880. The right or injured eye was somewhat atrophied; the tension was greatly diminished and the eye was slightly tender on pressure, although not markedly so. In the left eye there was photophobia, lachrymation, slightly sluggish pupil, and supra-orbital neuralgia.

Vision for distance tolerably good, V=15-xx; but it was quite impossible for him to read even Jaeger 11 for more than a minute or two, on account of the lachrymation and ciliary neuralgia which it induced.

Ophthalmoscopic examination showed well marked nuero-retinitis. Immediate enucleation of the injured eye was advised, but as it apparently gave him no little trouble, being only slightly painful at times, and as he was a farmer, he did not like the idea of wearing an artificial eye and submitting to all the inconveniences such an eye entails, he declined to submit to the operation and returned home. In the latter part of August, five or six weeks after his first visit, he returned to the city and was quite willing to submit to the enucleation of the injured eye as his symptoms had grown much worse after returning home. The tenderness, on pressure, of right eye was much increased, and the eye-ball was intensely injected. In the left eye there was considerable pain, supra-orbital neuralgia, sluggish pupil and photophobia V=10-xx.

August 20th, 1880. The patient was advised to remain quietly in a darkened room, and to take the following:

℞ Pulv. Dover.................................... 2.00
Quin. Sulph.................................... 3.00
Div. in parts, 20.
S. One every 4 hrs.

August 24th. The inflammatory symptoms were not so great. Ether was administered and the injured eye-ball removed, although with some difficulty, owing to its atrophied condition and some fibrous adhesions which completely surrounded it.

August 25th. Patient fully recovered from the effect of the ether, and the ciliary neuralgia less than it had been for months. Ordered Pil. Proto iodide Hyd. gr. ¼, one, three times daily.

September 10th. Wound nearly healed, neuralgia entirely absent. Vision much improved (15-xx) and patient allowed to return home, but advised to take the following alteration for six weeks or two months:

| | | |
|---|---|---|
| ℞ | Pot. Iodid.................................... | 16.00 |
| | Cinch. T. R.................................... | 20.00 |
| | Sarsar. Co. Tinc.................................... | 14.00 |

S. A teaspoonful, three times daily in water, after meals.

December 4th, 1880. The patient returned to the city for an artificial eye. There had been no recurrence of neuralgia, and vision fully restored (7-xx). The eye was placed in Müller's fluid for several weeks, then absolute alcohol, and after three months it was partially divided in its equatorial diameter. The vitreous was liquified and immediately escaped, leaving the retina in the form of a cord, extending from the fundus or posterior portion of the ball to the anterior, where it expanded, funnel-like, and enclosed a calcarious lens. The ossification was situated in the choroid, its thickest portion corresponding with the entrance of the optic nerve. The eye-ball was somewhat atrophied, measuring a trifle over two centimeters in the antero-posterior diameter, and a little less than two centimeters in its equatorial axis. The ossification measured fifteen mm. in length concavo—convex—the concavity looking forward and from four to eight mm. in width. Measured from the optic disc, it was a few mm. longer on the outer than on the inner side; its greatest thickness, which was at the optic disc, was two mm. The posterior of convex surface of the bone was well marked with choroidal pigment. Five years have now elapsed since the enucleation, and no relapse has occurred in the eye sympathetically affected.

The points of special interest in the case are the slight tenderness in the eye, primarily affected; the rapid and complete recovery and restoration of perfect vision after the injured eye had been removed. The question arises, if in cases of sympathetic ophthalmia, neuro-retinitis is the predominant symptom, is not the prognosis more favorable than when the cyclitis predominates? Which nerves are principally concerned in the former and which in the latter?

The case is certainly confirmatory of the conclusion arrived at by Knapp (Archives of Ophthalmology and Otology, Vol. II., p. 34), that "the diseases which lead to ossific productions are chronic inflammations of the interior coats of the eye called internal ophthalmia by the earlier, irido-choroiditis by modern, writers."

Dr. F. C. Hotz then read a paper entitled: *A case of Choroiditis following Typhoid fever.* He called attention to the fact that text-books record as sequelæ of this fever only corneal ulcers and abscesses, disturbances in the muscular apparatus, as paresis of accommodation and sphincter pupillæ, and paralysis of one or the other of the external muscles of the eyeball and affections of the optic nerve. Affections of the uveal tract are not even mentioned, this apparent immunity of the choroid to the effects of typhoid fever appears very remarkable if we consider the fact that other acute infectious diseases have been known at times to cause serious disturbances in this tunic. Cerebro-spinal-meningitis and relapsing fevers are known to cause respectively exudation into the vitreous, with subsequent atrophy of the globe and deposits on Descemet's membrane with posterior synechiæ (cyclitis and iritis). Similar changes may follow typhoid fever as was shown by the case cited.

Another interesting feature of the case was the rapid improvement which followed the hypodermic injections of pilocarpine.

A robust young farmer, aged sixteen, consulted Dr. Hotz on January 13th, with reference to diminished vision of the left eye. During recovery from an attack of typhoid fever, in October, he first noticed dimness of sight, which finally became entirely obscured. No syphilitic taint. At the time of examination the following was noted: Right eye V = 20–XX. em, normal fundus. Left eye, counted fingers at twelve feet; there was no pain, tenderness or redness. Transparent media, with exception of vitreous, clear. Pupil slightly enlarged, a mydriatic had not been used. Tension normal. Numerous floating opacities in the vitreous allowing, however, examination of fundus and discovery of a large whitish exudation in the periphery of the upper nasal section.

The patient was given Pot. Iod. gr. V. three times per diem. He returned February 5th. While at home the eye inflamed (evidently iritis) during two weeks. The brown pigment, dots upon the anterior capsule of lens, outlining size of the pupil were the same as on first examination. The pupil was larger and vitreous, so cloudy that but a faint reflex could be obtained with ophthalmoscope. He could barely count fingers at one foot. The patient was put under a treatment of hypodermic injections of gr. 1–6 of pilocarpine. After five injections, administered in one week, the vitreous was much clearer. V. had risen to 20–L, but patient felt so weakened from effects of remedy that he insisted on a short intermission. During this time sight grew worse and sank in one week to 20–C:

Descemet's membrane became dusty and vitreous cloudy again.

Pilocarpine treatment raised V. to 20–L; vitreous cleared up, ophthalmoscope showed a number of disseminated white patches in upper nasal section of choroid. From this time on the eye improved constantly. March 28th, very few opacities in vitreous, V = 20–XXX. May 21st, V = 20–XX, vitreous clear. In the upper nasal periphery of fundus numerous patches of choroidal atrophy.

